# Evolved increases in running performance in cold hypoxia in high-altitude deer mice

**DOI:** 10.1242/jeb.252284

**Published:** 2026-04-09

**Authors:** Derek A. Somo, Sophia C. Marangozis, Mei Le L. Cumming, Grant B. McClelland, Graham R. Scott

**Affiliations:** Department of Biology, McMaster University, Hamilton, ON, Canada, L8S 4L8

**Keywords:** High elevation, Environmental adaptation, Acclimatization, Exercise, Skeletal muscle, Mammal

## Abstract

The cold and hypoxic conditions at high altitude can challenge the ability of small endotherms to meet the high energy demands of locomotion and thermoregulation. We examined how high-altitude natives overcome this challenge through plastic and/or evolved improvements in locomotory performance. Deer mice (*Peromyscus maniculatus*) native to high and low altitude were born and raised in captivity, then acclimated to warm normoxia or cold hypoxia as adults. Running endurance was then measured in both warm normoxia and cold hypoxia across groups. Among mice acclimated to warm normoxia, endurance was greater in highlanders compared with lowlanders. Acclimation to cold hypoxia increased endurance in lowlanders, partially approaching values in highlanders. Body temperature declined while running in cold hypoxia in lowlanders, but highlanders were better at avoiding such declines. Our data suggest that evolved changes in thermoregulatory ability and muscle phenotype combine to improve locomotory performance in cold hypoxia in high-altitude deer mice.

## Teaser

**Summary:** High-altitude deer mice have greater running endurance and better maintain body temperature in cold hypoxia compared with low-altitude mice.

## INTRODUCTION

High-altitude environments pose significant challenges to homeostasis and performance in endotherms. Cold temperatures in these environments amplify the metabolic demands of thermogenesis, while hypoxia constrains the O_2_ supply to power key aerobic activities such as thermogenesis and locomotion. This metabolic challenge is amplified in small-bodied endotherms, whose high surface area to volume ratios exacerbate heat loss and increase demands for thermogenesis. Accordingly, small endotherms that are native to high altitude provide a powerful opportunity to understand the mechanistic underpinnings of environmental adaptation (McClelland and Scott, 2019; Monge and Leon-Velarde, 1991; Storz and Cheviron, 2021).

Deer mice (*Peromyscus maniculatus*) have provided appreciable insight into phenotypic plasticity and local adaptation in high-altitude environments. This species is widespread and has the broadest elevational distribution of any North American mammal, with permanent successful populations reaching the peaks of many of the continent's highest mountains (Avise et al., 1979; Hock, 1964; Natarajan et al., 2015; Snyder et al., 1982). At these high altitudes, a high aerobic capacity for thermogenesis has been shown to improve survival and has thus been linked to fitness (Hayes and O'Connor, 1999). Plastic responses to cold and/or hypoxia during early development and adulthood increase thermogenic capacity (Chappell et al., 2007; Hammond et al., 2002; Robertson and McClelland, 2021; Van Sant and Hammond, 2008). Overlaid upon this plasticity, high-altitude populations have evolved greater thermogenic capacity in hypoxia than their low-altitude counterparts (Cheviron et al., 2012; Tate et al., 2017). This variation in thermogenic capacity is supported by plastic and evolved changes in cardiorespiratory function, metabolic fuel transport and the phenotype of thermogenic tissues (Coulson et al., 2021; Ivy et al., 2021; Lyons and McClelland, 2022, 2024; Tate et al., 2020).

How plasticity and local adaptation to cold hypoxia shape locomotory performance in deer mice is not as well understood. Within high-altitude populations, thermogenic capacity is positively associated with above-ground activity (Sears et al., 2006), suggesting that locomotory performance requires a strong capacity to thermoregulate. Furthermore, the important role of oxidative muscles for both shivering thermogenesis and prolonged locomotion could underlie correlated variation in these traits in some situations. Indeed, highland deer mice have evolved a more aerobic muscle phenotype than their lowland conspecifics, characterized by greater oxidative fibre density, mitochondrial content and respiratory capacity, and oxidative enzyme activity (Dawson and Scott, 2022; Garrett et al., 2024; Lui et al., 2015; Mahalingam et al., 2017, 2020; Scott et al., 2015). However, cold hypoxia exposure may lead to conflicting demands between thermogenesis and locomotion (Chappell and Hammond, 2004), particularly if non-shivering thermogenesis by brown adipose tissue limits the availability of oxygen and metabolic fuels for the locomotory muscle, which could thus limit the capacity for sustained (endurance) locomotion in cold hypoxia. Constraints on locomotion in cold hypoxia are likely detrimental, given that locomotion underlies many fitness-relevant behaviours (foraging, predator evasion, finding mates, etc.), but it remains unknown whether highland deer mice mitigate such constraints.

Our objective was to shed light on this issue by examining plastic and evolved changes in the capacity for sustained locomotion in high-altitude deer mice. Populations of deer mice native to high and low altitude were born and raised in captivity, and adults from both populations were acclimated to warm normoxia or cold hypoxia in a full factorial design. Running endurance capacity was then measured using an incremental speed test on a treadmill in both warm normoxic and cold hypoxic test conditions. Body temperature was simultaneously monitored using passive integrated transponder (PIT) tags. We also performed an exploratory analysis of force at grip failure to assess whether previously observed differences in muscle phenotype might be associated with variation in other aspects of limb function. We hypothesized that acclimation to cold hypoxia would enhance running endurance in low-altitude mice, but that high-altitude mice would exhibit even greater running endurance in association with better maintenance of body temperature during sustained locomotion.

## MATERIALS AND METHODS

### Animals and acclimation conditions

Wild deer mice, *Peromyscus maniculatus* (J. A. Wagner 1845), were live trapped at low altitude near Kearney, NE, USA (Buffalo County, 40°41′58″N, 99°04′53″W; approximately 660 m above sea level) and at high altitude on the summit of Mount Blue Sky, CO, USA (formerly ‘Mount Evans’; Clear Creek County, 39°35′18″N, 105°38′38″W; 4350 m above sea level). Mice were transported to McMaster University (Hamilton, ON, Canada; 90 m above sea level) and bred in captivity within their respective populations to produce first generation progeny. Mice were housed in standard mouse cages (containing 7090 Teklad SaniChips^®^ animal bedding; Envigo, Indianapolis, IN, USA) under common laboratory conditions (∼22°C, ambient O_2_ ∼20 kPa, 12 h:12 h light:dark cycle) with rodent chow (Teklad 22/5 Rodent Diet formula 8640; Envigo) and water available *ad libitum*. After reaching ≥6 months of age, mice either continued to be held in common laboratory conditions or were acclimated to cold hypoxia (5°C, 12 kPa O_2_; simulating conditions at Mount Blue Sky) for 6 weeks prior to use in experiments. To achieve cold hypoxia, hypobaric chambers (described previously in Lui et al., 2015; Mahalingam et al., 2017; McClelland et al., 1998) were placed in a temperature-controlled room at the target temperature. All conditions and experimental procedures were approved by the McMaster University Animal Research Ethics Board according to guidelines from the Canadian Council on Animal Care under AUP 23-85.

### Implantation of thermosensitive passive transponders

One week prior to whole-animal testing, thermosensitive PIT tags (BioTherm13 PIT tag, BioMark, Biose, ID, USA) were implanted intraperitoneally using a sterilized trochar (BioMark) to monitor body temperature. Mice were lightly anaesthetized using isoflurane (∼3–4% in O_2_) during implantation, a procedure that took ∼1–2 min, and were monitored afterwards to ensure full recovery prior to endurance and strength testing.

### Experimental protocol overview

Each mouse was tested for both treadmill running endurance and force at grip failure over a 2 week period. On day 1, force at grip failure measurements were taken, followed by the first of three treadmill training sessions. Subsequent training sessions were conducted on days 2 and 3 and at similar times of day. Forty-eight hours after the final treadmill training session, a treadmill endurance test was performed in warm normoxia (22°C, ∼20 kPa O_2_). A second endurance test in warm normoxia was performed 72 h later to assess repeatability. A final endurance test was then performed another 72 h later in acute cold hypoxia (5°C, 12 kPa O_2_). Force at grip failure was again measured 24 h later. Measurements of force at grip failure were only made in warm normoxia because of the practical limitations of using the test apparatus in cold hypoxia. After all measurements were complete, mice were euthanized by isoflurane overdose followed by cervical dislocation.

### Treadmill running endurance

Endurance running capacity was measured following established protocols that have been described previously (Eizenga et al., 2023; Meek et al., 2009), but with minor modifications. Mice were run on a Plexiglas-enclosed rodent treadmill (compartment dimensions of 384 mm×51 mm×102 mm; Columbus Instruments, Columbus, OH, USA) at an incline of 10 deg. Prior to endurance testing, mice were trained to run on the treadmill over the course of three training sessions that were scheduled as described above. For each run, mice were introduced to the chamber for 10 min in the dark by covering the chamber with a cloth. The cloth was then removed when the treadmill was started so mice ran in the light. In training session 1, mice were run at 10 m min^−1^ for 15 min. In training session 2, mice were run at 10 m min^−1^ for 1 min then 14 m min^−1^ for 14 min. In training period 3, mice were run at 10 m min^−1^ for 1 min, 14 m min^−1^ for 1 min, then 18 m min^−1^ for 13 min. To test endurance running capacity, mice were run throughout the following protocol of incremental speed increases: 10 m min^−1^ for 2 min, 14 m min^−1^ for 2 min, 18 m min^−1^ for 2 min, 20 m min^−1^ for 2 min, and then increasing speeds by 1 m min^−1^ every 2 min thereafter, up to a maximum of 69 m min^−1^ (the maximum speed of the treadmill). For the modest number of mice that reached this maximum speed, we maintained this maximum speed until they reached exhaustion. In warm normoxic trials, a fan at the front of the treadmill pulled room air into the chamber to maintain the supply of fresh normoxic air. Cold hypoxia trials took place in an environmental chamber set to 5°C and the hypoxia level was maintained using an O_2_ fuel cell sensor (FC-1S Oxygen Battery, Sable Systems International, North Las Vegas, NV, USA) placed inside the treadmill and connected to a ROXY-4 gas regulator (Sable Systems International). The ROXY-4 uses the reading from the O_2_ sensor as a feedback signal to control the flow of N_2_ (from a compressed N_2_ tank) into the treadmill chamber using a solenoid valve, balanced against a consistent flow of room air entering the chamber through a small port, and thus maintaining O_2_ partial pressure at 12 kPa. The oxygen sensor was calibrated daily at experimental temperature. Running trials were conducted during daylight hours (09:00–19:00 h local time).

Body temperature (*T*_b_) was recorded non-invasively at the end of each 2 min period using a PIT tag reader (Global Pocket Reader Plus, Destron Fearing, DFW Airport, TX, USA). As mice approached exhaustion, visual and audible cues (e.g. tapping on the walls of the treadmill, shining flashlight, etc.) were used as motivation. Mice were considered exhausted when they were unable to run on the treadmill for 4 consecutive seconds. Mice were then removed from the treadmill and placed in a darkened chamber to recover for 15 min before being returned to their home cage.

### Force at grip failure

Force at grip failure was measured using a metal grid (16 cm×10 cm) attached to a force transducer and meter (BIO-GS3 Grip Strength Test, Bioseb, Pinellas Park, FL, USA) following the manufacturer’s instructions. A mouse was held by the tail and gently lowered onto the grid, allowing the mouse to grab hold with all four limbs. The tester then gently and steadily pulled the tail of the mouse in the opposite direction to the transducer until the mouse's grip failed and the resulting force (in grams) at failure was recorded. Initial testing found that vertically orienting the test apparatus and pulling the mice upwards resulted in the most successful trials in which all four limbs were used to grip the grid, so this orientation was used for all force at grip failure data here. The test was repeated 5 times per mouse, with ∼5 min rest in between tests, both before (‘initial force at grip failure’) and after completion of the endurance trials (‘final force at grip failure’), and the average values of each series of tests is reported.

### Statistical analysis

Statistical analyses were performed in R (version 4.4.0, https://www.r-project.org/). Body mass statistics and sample sizes are reported in Table S1. We calculated repeatability using the rptR package (Stoffel et al., 2017) for endurance time and total distance run in warm normoxic conditions between endurance trials 1 and 2, as well as average force at grip failure between the initial and final force at grip failure tests. Overall repeatability for each trait was calculated while controlling for the effects of population (lowland versus highland), acclimation (warm normoxia versus cold hypoxia) and their potential interaction. Standard error was quantified via 1000 bootstrap iterations and *P*-values were calculated using likelihood ratio tests (LRT) using default package settings (Stoffel et al., 2017).

The fixed effects of population (lowland or highland), acclimation environment (warm normoxia or cold hypoxia) and test condition (warm normoxic or cold hypoxic) on endurance time and total distance run data were tested using linear mixed-effects models using the ‘lmer’ function in the ‘lmerTest’ package (Kuznetsova et al., 2017). Models also included the random effect of mouse identity to account for repeated measurements across test conditions. The greater endurance time from the two warm normoxic trials was generally used as the warm normoxic endurance value for each mouse. However, we were unable to complete both warm normoxic trials for a small subset of mice, for which we chose to include the data from the single completed trial (this seemed reasonable given the relatively high repeatability of endurance in warm normoxia). Similarly, we were unable to complete a cold hypoxic trial for a small subset of mice that were run in warm normoxia. Given that linear mixed effects models are robust to small amounts of missing data, these mice were included in analyses of endurance and total distance run.

We used a generalized additive mixed model to test the effects of population, acclimation, acute test condition, body mass and sex on body temperature during endurance running trials using the ‘gam’ function in the R package ‘mgcv’ (Wood, 2011). Smoothing curves were fitted for the main effects as well as for the random effect of individual. To facilitate comparisons of the effect of time spent running on body temperature, we calculated ω^2^ effect sizes from approximate *F*-statistics and effective degrees of freedom of the smoothing curves following Wood (2013).

The fixed effects of population (lowland or highland) and acclimation environment (warm normoxic or cold hypoxic) on force at grip failure were tested using standard linear models using the ‘lm’ function. The average final force at grip failure value for each mouse was used in this analysis because mean final force at grip failure tended to be higher than mean initial force at grip failure (paired *t*-test, *P*=0.085) and because the repeatability of force at grip failure was low (Fig. S1; *R*=0.112±0.119, estimate±s.e., *P*=0.324) and the final tests were completed closer to the time of the endurance tests.

The fixed effects of sex and body mass were also included in all linear mixed-effects models and standard linear models. Residuals from endurance time, total distance run and force at grip failure models were homoscedastic and normally distributed based on visual assessment of residuals versus fitted values plots and *Q*–*Q* plots, respectively. Because force at grip failure was found to be significantly affected by sex and body mass, we calculated partial residuals to adjust for these effects using the ‘visreg’ R package (Breheny and Burchett, 2017). These partial residuals are reported here to evaluate the effects of population (lowland or highland) and acclimation environment. We used Type II ANOVA to test for the significance of fixed effect model terms at α=0.05, with degrees of freedom in the linear mixed-effects models calculated using Satterthwaite's method. Estimated marginal means, which are presented along with the raw data, were calculated using the ‘emmeans’ R package (https://CRAN.R-project.org/package=emmeans). The emmeans package was also used for pairwise comparisons between populations or acclimation environments using Tukey's *post hoc* tests when significant fixed effects for these variables were detected.

## RESULTS AND DISCUSSION

**Fig. 1.**
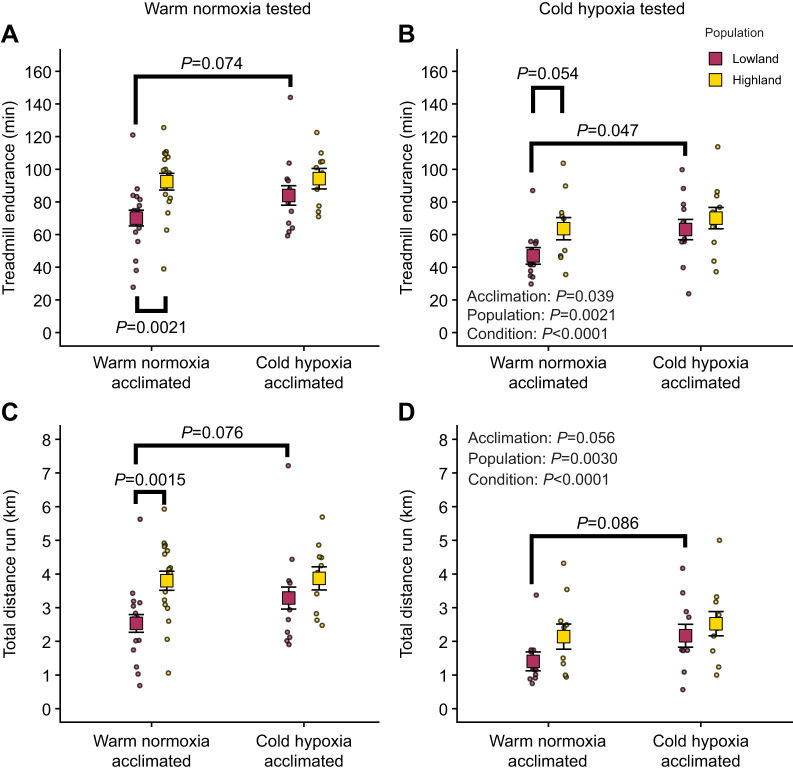
**Highland deer mice have greater running endurance than lowland deer mice.** Treadmill running endurance was measured as endurance time (A,B) and total distance run (C,D) in warm normoxic test conditions (A,C; 22°C, ∼20 kPa O_2_) and cold hypoxic test conditions (B,D; 5°C, 12 kPa O_2_). Measurements were performed in highland and lowland populations of deer mice that were born and raised in captivity and acclimated as adults to warm normoxia (∼22°C, ∼20 kPa O_2_) or cold hypoxia (5°C, 12 kPa O_2_). Raw data for individuals are shown as small circles; squares and error bars represent estimated marginal means ±standard error of the mean (s.e.m.) conditioned on endurance and distance univariate model terms (i.e. removing the variation associated with sex and body mass). ANOVA *P*-values are provided in B for the endurance time model and in D for the total distance model (see Table S1 for full ANOVA results and sample sizes). *Post hoc* comparisons were performed using the Tukey method.

Consistent with our prediction, highland deer mice had greater running endurance than lowland mice (Fig. 1; Table S1). Endurance time was generally repeatable (*R*=0.591; Fig. S1A), as was total distance run (*R*=0.609; Fig. S1B). Endurance time and distance were significantly affected by population (*P*=0.0021 and 0.0030), acclimation environment (*P*=0.039 and 0.056) and test condition (both *P*<0.0001; Table S2). Among warm normoxia-acclimated mice, highlanders ran on average ∼22 min longer (Fig. 1A) and ∼1.3 km farther (Fig. 1C) than lowlanders in warm normoxic test conditions and ∼17 min longer (Fig. 1B) and ∼700 m farther (Fig. 1D) in cold hypoxic test conditions. Acclimation of lowland mice to cold hypoxia increased running endurance in both warm normoxic (∼14 min longer, ∼760 m farther) and cold hypoxic test conditions (∼16 min longer, ∼760 m farther) to partially approach the endurance of highlanders. Endurance did not change significantly with cold hypoxia acclimation in highlanders, but endurance time and distance in cold hypoxia increased slightly on average.

Running endurance is generally determined by aerobic capacity (*V̇*_O_2_,max_), cost of transport (COT) and lactate threshold (Bassett, 2000). In deer mice, highlanders acclimated to warm conditions had greater exercise *V̇*_O_2_,max_ than lowlanders when tested in hypoxia, but not when tested in normoxia (Lui et al., 2015). However, COT at submaximal running speeds equivalent to ∼75% *V̇*_O_2_,max_ was lower in highlanders than in lowlanders, by ∼24% in normoxia and ∼14% in hypoxia (Lau et al., 2017). Therefore, increases in exercise *V̇*_O_2_,max_ and/or reductions in COT may contribute to improving endurance running capacity in highland deer mice. The lactate threshold of deer mice is unknown, but evidence suggests that blood lactate concentrations during submaximal exercise (15 min of 75% or 80% of *V̇*_O_2_,max_) is lower in some conditions in highlanders compared with lowlanders (Coulson et al., 2024; Dessureault et al., 2024; Lau et al., 2017). Muscle oxidative capacity influences each of these traits, so the evolved increase in oxidative capacity in highlanders likely contributes to their increased running endurance among mice acclimated to warm normoxia.

The effects of cold hypoxia acclimation on endurance running capacity that we observed in lowlanders could be explained, at least in part, by effects of cold acclimation on muscle phenotype. In white-footed mice (*Peromyscus leucopus*), for example, acclimation to cold or cold hypoxia increases oxidative fibre density and mitochondrial respiratory capacity of the gastrocnemius (Mahalingam et al., 2020). Similarly, acclimation of rats and guinea pigs to cold and/or cold hypoxia has been observed to increase muscle oxidative capacity and capillarity (Banchero et al., 1985; Harri and Valtola, 1975; Jackson et al., 1987; Sillau et al., 1980). These plastic adjustments in muscle phenotype are likely a response to the increased aerobic demands for shivering thermogenesis, associated with increases in routine heart rate and food consumption in cold hypoxia (Wearing and Scott, 2022), and could lead to associated improvements in aerobic running performance. In contrast, acclimation to cold hypoxia has very little effect on the oxidative capacity of the gastrocnemius in highlanders (Lyons and McClelland, 2022; Mahalingam et al., 2020), consistent with the lack of plasticity in endurance capacity. However, exercise *V̇*_O_2_,max_, running COT and fuel selection during exercise have yet to be measured after cold hypoxia acclimation in deer mice.

**Fig. 2.**
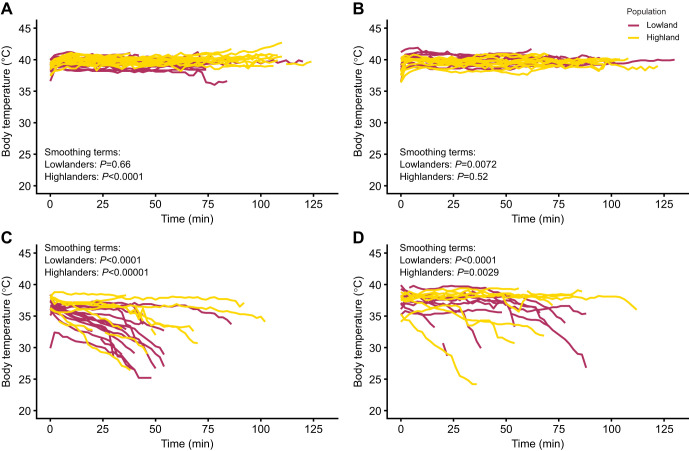
**Highland deer mice better maintain body temperature than lowland deer mice during endurance running in cold hypoxia.** (A) Warm normoxia acclimated, warm normoxia tested (*N*=15 highlanders, *N*=17 lowlanders). (B) Cold hypoxia acclimated, warm normoxia tested (*N*=10 highlanders, *N*=11 lowlanders). (C) Warm normoxia acclimated, cold hypoxia tested (*N*=8 highlanders, *N*=15 lowlanders). (D) Cold hypoxia acclimated, cold hypoxia tested (*N*=8 highlanders, *N*=9 lowlanders). Body temperature was measured every 2 min using intraperitoneal passive integrated transponder (PIT) tags during the running endurance tests shown in Fig. 1 and was modelled using a generalized additive model (GAM). The smoothing terms from the GAM were used to fit the effects of time on body temperature during running tests in each group. The *P*-values for the smoothing curves for each population, acclimation and condition group are reported in their respective panels, and full GAM model results are reported in Table S3. Note, some sample sizes (*N*) are slightly lower than those in Fig. 1 because of a failure of PIT tags during or prior to some trials, precluding body temperature measurement.

In the warm normoxic test condition, *T*_b_ rose at the start of running trials but nearly all mice maintained relatively constant *T*_b_ thereafter until exhaustion (Fig. 2A,B), as previously observed (Eizenga et al., 2023). This early rise in *T*_b_ was associated with significant or nearly significant smoothing terms during the endurance tests in warm normoxia (Table S3; note that the smoothing terms describe the effects of time in the generalized additive model). In contrast, *T*_b_ declined in many mice during endurance tests in cold hypoxia (test condition effect, *P*<0.0001) (Fig. 2D,E). However, highlanders tended to better maintain *T*_b_ than lowlanders while running in cold hypoxia. In particular, among mice acclimated to cold hypoxia, 5 out of 9 highland mice maintained *T*_b_ while running in cold hypoxia (<2°C variation) but no lowland mice were able to do so. This difference was supported by the ω^2^ effect sizes for the smoothing terms from the generalized additive model, which showed an approximately 14-fold greater effect of cold hypoxia in lowlanders (ω^2^=0.04) than in highlanders (ω^2^=0.003) (Table S3). The effect sizes were generally greater among mice acclimated to warm normoxia, but there was still a large difference between lowlanders (ω^2^=0.207) and highlanders (ω^2^=0.018). Therefore, even though cold hypoxia acclimation increased running endurance in lowlanders to approach the values in highlanders, lowlanders still experienced more intense hypothermia. Chappell and Hammond (2004) have suggested that falling *T*_b_ during intense running in severe cold could result from two non-exclusive causes: (i) conflicts between the locomotory and thermogenic functions of skeletal muscle; and/or (ii) supply limitations to some other thermoeffectors (e.g. brown adipose tissue, BAT) as a result of the high O_2_ and fuel demands of locomotory muscles. In support of the latter possibility, highland deer mice can maintain higher cardiac output at thermogenic *V̇*_O_2_,max_ than lowland mice (Tate et al., 2020), and may better avoid O_2_ and fuel supply limitations to thermogenic tissues. By contrast, lowlanders may need to reduce perfusion to BAT during intense locomotion and thus suffer greater declines in *T*_b_. The ability to maintain *T*_b_ during locomotion is likely critical for activity, foraging and fitness in highland deer mice in the wild.

**Fig. 3.**
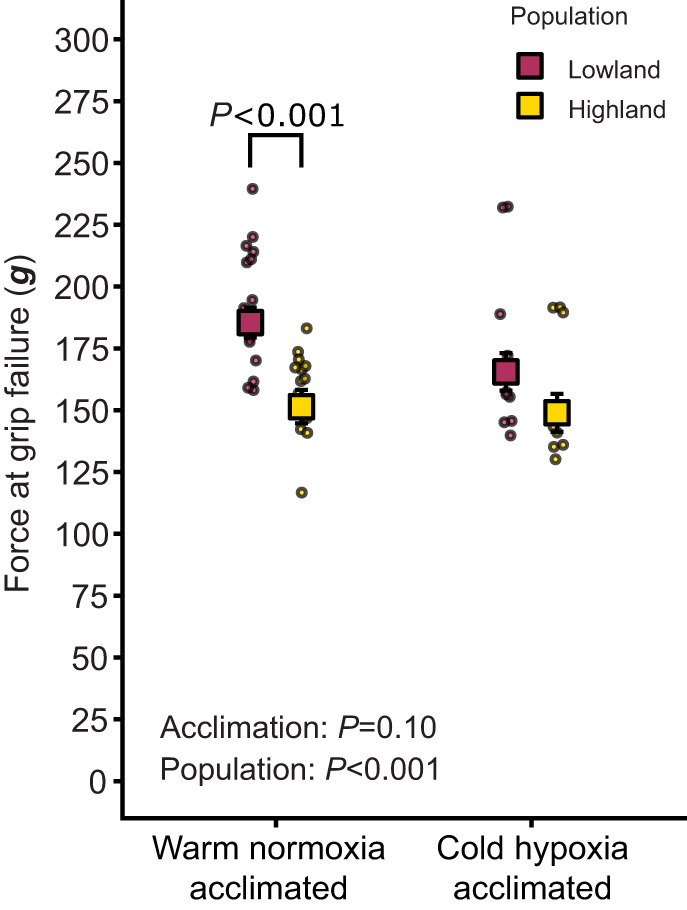
**Increases in running endurance are associated with reductions in force at grip failure.** Force at grip failure was measured under warm normoxic conditions in highland and lowland populations of deer mice that were born and raised in captivity and acclimated as adults to warm normoxia (∼22°C, ∼20 kPa O_2_) or cold hypoxia (5°C, 12 kPa O_2_). The force at grip failure values reported here are partial residuals (extracted from the linear model) that adjusted for the significant effects of body mass and sex (Fig. S2, Table S1). Small circles represent individual data and squares and error bars represent means±s.e.m. Sample sizes are in Table S1.

We also performed an exploratory analysis of force at grip failure to assess whether there might be variation in other aspects of limb muscle function across groups. This measure is a simple and commonly used marker of limb muscle strength (Huang et al., 2023; Owendoff et al., 2023), although there can be some technical variation due to the challenges of accurately controlling the rate of loading. Force at grip failure was lower in highlanders compared with lowlanders (Fig. 3; Table S2), as reflected by a significant population effect on this trait (*P*=0.00036). Force at grip failure declined slightly in lowland mice after cold hypoxia acclimation, halving the difference between populations from ∼34 g to ∼17 g (Fig. 3). Force at grip failure was higher in females and increased with body mass, but neither factor impacted running endurance or total distance run (Table S2, Fig. S2).

When the variation in running endurance and force at grip failure is considered together, a potential common explanation is the previously observed variation in muscle phenotype. Specifically, highlanders generally have smaller gastrocnemius muscles with more oxidative and less glycolytic fibres compared with lowlanders (glycolytic fibres are larger than oxidative fibres in deer mice) (Lui et al., 2015). These population differences in fibre-type composition are associated with an enhanced biochemical capacity for oxidative metabolism and elevated mitochondrial volume (Dawson and Scott, 2022; Lui et al., 2015; Mahalingam et al., 2017, 2020; Scott et al., 2015), and similar variation appears to exist in other locomotory muscles across the body (Garrett et al., 2024). These population differences in muscle phenotype and size likely contribute to improving muscle endurance but reducing strength. Furthermore, acclimation to cold hypoxia in lowlanders leads to a shift in fibre-type composition to more oxidative and less glycolytic fibres, approaching but not reaching the oxidative fibre densities of highlanders (Mahalingam et al., 2020), which could similarly alter muscle endurance and strength. When also considering that the oxidative capacity and fatigue resistance of locomotory muscles are important determinants of prolonged shivering, it is possible that directional selection on increased thermogenic capacity at high altitude (Hayes and O'Connor, 1999) led to correlated changes in running endurance and muscle strength in high-altitude deer mice.

Overall, our findings suggest that plastic and evolved changes in high-altitude deer mice help improve running performance and body temperature maintenance in cold hypoxia, but are associated with reductions in limb strength. The potential ecological costs of these changes are unclear. High-altitude mice must still compete with conspecifics and avoid predation, tasks that may be impaired by a loss of strength. However, it is possible that such biotic factors are less of an issue at high altitude, such that plastic and evolved changes in muscle phenotype, endurance and aerobic performance take precedence to cope with the harsh abiotic environment. When considering that many highland taxa exhibit more oxidative muscles than their lowland counterparts, our results suggest that correlated changes in shivering thermogenesis, locomotory endurance and/or strength may have arisen across other high-elevation taxa.

## Supplementary Material

10.1242/jexbio.252284_sup1 Supplementary information
