# Supplementary material for: Evolved increases in running performance in cold hypoxia in high-altitude deer mice
Source: J Exp Biol. 2026 Apr 9;229(7):jeb252284. doi: 10.1242/jeb.252284 (PMC13091496; doi:10.1242/jeb.252284)
Supplement: Supplementary information [file jexbio-229-252284-s1.pdf]

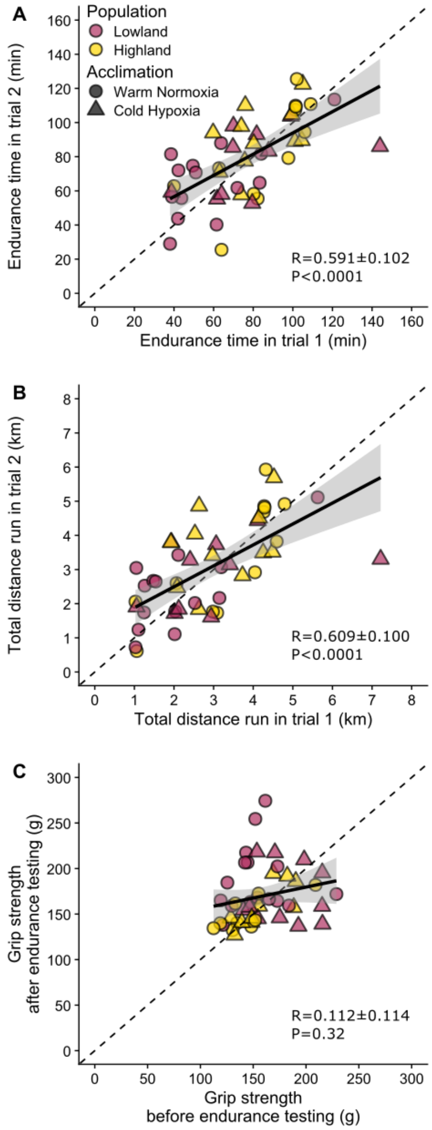

Fig. S1. Repeatability of running endurance, total distance run, and force at grip failure. Visualization of the repeatability of running endurance time (A), total distance run during endurance tests (B), and average force at grip failure measured before and after the 2 week endurance testing protocol (C). Repeatability was calculated using the "rptR" R package (Stoffel et al., 2017) and the repeatability model for each trait included effects of acclimation, population, and their interaction. Repeatability (R) and the P value for repeatability are shown on each respective panel.

Journal of Experimental Biology • Supplementary information

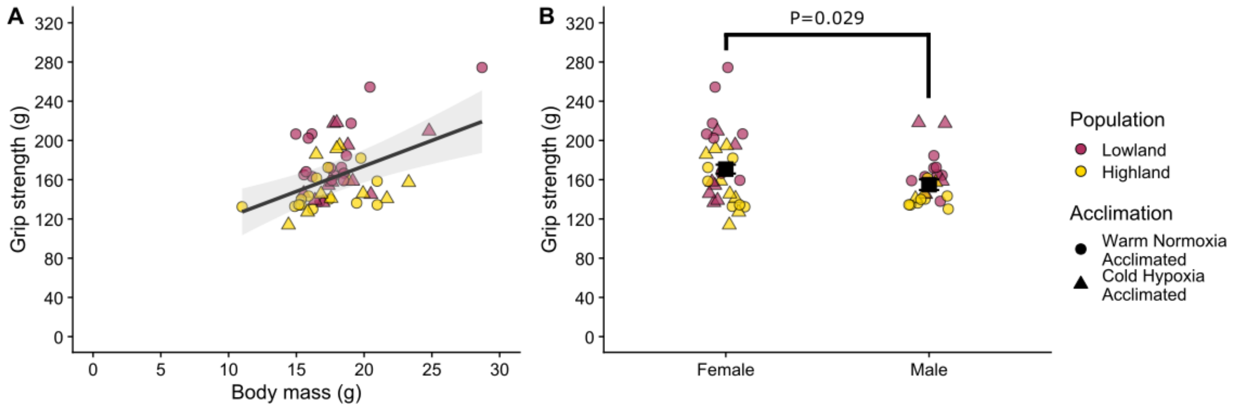

Fig. S2. Force at grip failure was affected by both body mass (A) and sex (B). The regression line and 95% confidence interval plotted in (A) is the global effect of body mass on force at grip failure averaged over the effects of acclimation, population, and sex. The black squares and error bars in (B) are estimated marginal means ± s.e.m. conditioned on the force at grip failure linear model terms. P-values on each panel are for the associated fixed effects from ANOVA for the linear model of force at grip failure (full model results are reported in Table S2). Sample sizes for grip strength *vs*. body mass (A): WNac highlanders, N=15; WNac lowlanders, N=17; CHac highlanders, N=10; CHac lowlanders, N=11.

Table S1. Body masses and sample sizes for treadmill running endurance and force at grip failure tests.

	High-altitude population				Low-altitude population			
Acclimation	Warm normoxia		Cold hypoxia		Warm normoxia		Cold hypoxia	
Test condition	Warm normoxia	Cold hypoxia	Warm normoxia	Cold hypoxia	Warm normoxia	Cold hypoxia	Warm normoxia	Cold hypoxia
Endurance tests	17.4±3.40 (16)	16.6±2.56 (9)	19.1±3.29 (11)	19.4±2.55 (10)	18.0±3.18 (18)	18.2±3.37 (16)	19.0±2.82 (12)	18.9±2.91 (11)
Force at grip failure tests	16.9±2.73 (14)	-	18.3±2.58 (11)	-	18.0±3.13 (17)	-	18.4±2.50 (11)	-

Body masses (g) are presented as means±s.e.m. followed by sample sizes in parentheses. Average body masses and sample sizes differ between measures and test conditions because not all mice completed all warm normoxic and cold hypoxic endurance trials or force at grip failure tests (see Methods and Materials for additional details).

Table S2. ANOVA results for linear models.

		Body mass	Sex	Population (Pop)	Acclimation (Acc)	Condition (Cond)	Pop×Acc	Pop×Cond	Acc×Cond	Pop×Acc×Cond	σ_{ID}|σ_{Resid}
Endurance Time	F	$F_{1,53.2}$=0.0251	$F_{1,48.8}$=0.29	$F_{1,49.1}$=10.6	$F_{1,48.9}$=4.51	$F_{1,46.1}$=50.3	$F_{1,49.3}$=1.41	$F_{1,47.1}$=0.441	$F_{1,45.9}$=0.22	$F_{1,46.2}$=0.044	134|285
	P	0.87	0.59	**0.0021**	**0.039**	**<0.001**	0.24	0.51	0.65	0.84	
Total Distance	F	$F_{1,53.0}$=0.0509	$F_{1,48.6}$=0.236	$F_{1,48.9}$=10.0	$F_{1,48.7}$=3.83	$F_{1,45.8}$=48.3	$F_{1,49.1}$=1.14	$F_{1,46.8}$=1.10	$F_{1,45.6}$=0.144	$F_{1,45.9}$=0.16	0.41|0.84
	P	0.82	0.63	**0.0027**	0.056	**<0.001**	0.29	0.30	0.71	0.69	
Force at grip failure	F	$F_{1,47}$=16.7	$F_{1,47}$=5.08	$F_{1,47}$=14.8	$F_{1,47}$=2.74	-	$F_{1,47}$=1.56	-	-	-	-
	P	**0.00017**	**0.029**	**0.00036**	0.10		0.22				

Endurance time and total distance during treadmill running tests were analyzed using linear mixed-effects models (including mouse ID as a random effect to account for repeated measures), and force at grip failure was analyzed using a standard linear model. Bold indicates significance at α=0.05. σ_{ID}, variance accounted for by repeated measures within individual mouse; σ_{Resid}, residual variance.

Table S3. ANOVA results for the generalized additive model of body temperature during endurance tests.

	$F_{d..f.}$	P	ω^2
Parametric terms			
Population	F_1=0.0220	0.88	< 0.001
Acclimation	F_1=1.56	0.21	< 0.001
Condition	F_1=3230	**<0.001**	0.482
Body mass	F_1=42.6	**<0.001**	0.012
Sex	F_1=0.179	0.67	< 0.001
Smoothing terms			
Time×LA/WNa/WNc	$F_{1.17}$=0.199	0.66	< 0.001
Time×HA/WNa/WNc	$F_{1.01}$=17.7	**<0.001**	0.005
Time×LA/CHa/WNc	$F_{3.32}$=3.90	**0.0072**	0.002
Time×HA/CHa/WNc	$F_{2.10}$=0.628	0.52	< 0.001
Time×LA/WNa/CHc	$F_{7.24}$=129	**<0.001**	0.207
Time×HA/WNa/CHc	$F_{4.74}$=17.4	**<0.001**	0.018
Time×LA/CHa/CHc	$F_{5.30}$=37.0	**<0.001**	0.044
Time×HA/CHa/CHc	$F_{4.62}$=3.78	**0.0029**	0.003
Individual	$F_{49.0}$=38.1	**<0.001**	0.338

Smoothing term abbreviations: LA, Lowlander; HA, Highlander; WNa, Warm Normoxia acclimated; CHa, Cold Hypoxia acclimated; WNc, Warm Normoxic test condition; CHc, Cold Hypoxic test condition. Note that degrees of freedom for smoothing terms are reference degrees of freedom for parametric terms and estimated degrees of freedom for the smoothing terms as reported in the model summary by the generalized additive model "mgcv" R package. Significant P values at α=0.05 are bolded. ω^2, effect size estimates of time spent running in cold hypoxia in the endurance trials.

Journal of Experimental Biology • Supplementary information
